# Whole-Genome Sequence of High Salt and Heavy Metal-Tolerant *Bacillus stratosphericus* Strain 5Co, Isolated from Lichen *Usnea florida* in Central Florida, United States

**DOI:** 10.1128/genomeA.00500-17

**Published:** 2017-06-15

**Authors:** Pushpa Lata, Subramaniam S. Govindarajan, Feng Qi, Jian-Liang Li, Santosh K. Maurya, Malaya K. Sahoo

**Affiliations:** a Department of Microbiology and Molecular Biology, University of Central Florida, Orlando, Florida, USA; b Analytical Genomics Core, Sanford Burnham Prebys Medical Discovery Institute at Lake Nona, Orlando, Florida, USA; c Applied Bioinformatics Core, Sanford Burnham Prebys Medical Discovery Institute at Lake Nona, Orlando, Florida, USA; d Sanford Burnham Prebys Medical Discovery Institute at Lake Nona, Orlando, Florida, USA; e Department of Pathology, Stanford University School of Medicine, Palo Alto, California, USA

## Abstract

*Bacillus stratosphericus* strain 5Co was isolated from lichen *Usnea florida* in central Florida, United States. Here, we report a draft genome sequence of this strain, which consists of 159 contigs spanning 3,628,496 bp, with a G+C content of 41.3% and comprises 3,729 predicted coding sequences.

## GENOME ANNOUNCEMENT

*Bacillus stratosphericus* is a Gram-positive endospore forming bacterium that belongs to phylum *Firmicutes*. It occupies quite distinct habitats, originally isolated from stratospheric air samples collected above 24 km of altitude and recently from the Mid-Atlantic-Ridge seafloor (5,500 m depth (1, 2). In this study, *Bacillus stratosphericus* strain 5Co was isolated from medicinally important fruticose lichen *Usnea florida* that grows luxuriously in the central Florida region of the United States. The *B. stratosphericus* strain 5Co exhibits a high level of tolerance to salt (15% NaCl) and heavy-metal cobalt (5 mM CoCl_2_). Here, we report the whole-genome sequence of *B. stratosphericus* strain 5Co. The genomic DNA of *B. stratosphericus* strain 5Co was isolated using a QIAamp DNA minikit (Qiagen, Germantown, MD), according to the manufacturer’s instructions. DNA quality was checked by a NanoDrop spectrophotometer (Thermo Scientific) and quantitated by a Qubit 2.0 fluorometer (Life Technologies) and Agilent 2100 Bioanalyzer (Agilent Technologies, Santa Clara, CA). The genomic DNA was fragmented and tagged with sequencing adapters in a single tube enzymatic reaction using the Nextera XT DNA library preparation kit (Illumina, San Diego, CA). The resulting library was sequenced using the Illumina MiSeq sequencing platform (Illumina, San Diego, CA) and generated 4.4 million reads. The reads were assembled using Minia version 2.0.3 with default parameters and a k-mer size of 121 (3). A total of 159 contigs were obtained and the *N*_50_ of the contigs was 106,700 bp. The maximum length of the contig was 315,691 bp with a total length of 3,628,496 bp genome. The G+C content of the genome was 41.3%. The protein-coding regions were predicted by NCBI Prokaryotic Genome Annotation Pipeline (release 2013; https://www.ncbi.nlm.nih.gov/genome/annotation_prok/). A total of 3,729 coding genes were identified in the 3.6 Mb genome. Seventy four tRNA genes, 16 rRNA (seven 5S, five 16S rRNAs, four 23S rRNAs), and five noncoding RNAs (ncRNAs) genes have putative functions assigned on the basis of the annotation. Cation efflux pump protein, copper resistance protein CopZ and arsenical efflux pump membrane protein ArsB were identified in the genome suggesting the strain's ability to tolerate higher concentrations of various heavy metals.

### Accession number(s).

This whole-genome shotgun project has been deposited at DDBJ/ENA/GenBank under the accession no. MWKO00000000. The version described in this paper is the first version, MWKO01000000.
